# Photoresponsive Delivery Microcarriers for Tissue Defects Repair

**DOI:** 10.1002/advs.201901280

**Published:** 2019-08-20

**Authors:** Xin Zhao, Yuxiao Liu, Changmin Shao, Min Nie, Qian Huang, Jieshou Li, Lingyun Sun, Yuanjin Zhao

**Affiliations:** ^1^ Department of Rheumatology and Immunology The Affiliated Drum Tower Hospital of Nanjing University Medical School Nanjing 210008 China; ^2^ Research Institute of General Surgery Jinling Hospital Medical School of Nanjing University Nanjing 210002 China; ^3^ Department of General Surgery The First Affiliated Hospital of Soochow University Suzhou 215006 China; ^4^ State Key Laboratory of Bioelectronics School of Biological Science and Medical Engineering Southeast University Nanjing 210096 China

**Keywords:** drug delivery, microcarriers, microfluidics, tissue defects, vascular endothelial growth factors (VEGFs)

## Abstract

Intelligent responsive microcarriers have emerged as a promising class of biomaterials for therapeutic delivery and tissue regeneration, since they can respond to external stimuli and release the loaded drugs in an active manner. Among various available stimuli, near‐infrared (NIR) light is particularly attractive because it can penetrate biotic tissues with sufficient intensity and minimal damage. In this work, a kind of photoresponsive delivery microcarriers (PDMs) is developed using microfluidics. The microcarriers consist of NIR‐absorbing graphene oxide, thermosensitive poly(*N*‐isopropylacrylamide), and biocompatible gelatin methacrylate. Under NIR light, the PDMs exhibit an evident volume shrinkage and effectively trigger the drug release. After the NIR light is switched off, the shrunken microcarriers return to their original size. This reversible process can be stably repeated for many cycles. An in vitro experiment demonstrates that the NIR‐radiated PDMs can actively release vascular endothelial growth factors and improve the tube formation of human umbilical vein endothelial cells. The results from the in vivo experiment also show an obvious photothermal effect and superior therapeutic efficacy of these PDMs in a rat model of tissue defects. These features make the PDMs an excellent drug delivery system and represent a great potential for clinical applications in tissue repair.

## Graphical abstract

**Photoresponsive delivery microcarriers** are developed using microfluidics, which consist of graphene oxide, poly(*N*‐isopropylacrylamide), and gelatin methacrylate. These microcarriers can actively respond to near‐infrared light and release the encapsulated drugs according to the irradiation frequency. After being implanted into a rat model of abdominal wall defects, they also exhibit potential value in tissue repair.

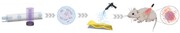

## 1 Introduction

Tissue defects caused by trauma, infection, congenital disease, or cancer resection have become a severe threat to human health.^1^, ^2^, ^3^ The self‐healing ability of human body is limited and the reasons are multifactorial, but one of the major causes is tissue ischemia.^4^, ^5^ It has been identified that vascular endothelial growth factors (VEGFs) can interact with the target receptors on endothelial cells (ECs), contributing greatly to vascular formation and tissue regeneration.^6^, ^7^ Consequently, improving hemodynamics via VEGFs administration in defected tissues is an emerging strategy to promote the healing of chronic wounds. However, the application of VEGFs or other active molecules/proteins is hampered by their rapid degradation. To address this problem, a number of investigators have employed various techniques to design microcarriers for biomacromolecules delivery, such as emulsification‐solvent evaporation, spray‐drying, and phase separation.^8^, ^9^ These techniques are associated with inherent drawbacks, especially the instability of biomacromolecules which are susceptible to the large shear force and strong oscillation during fabrication process. In addition, these techniques are also lack of precise control over the resultant microcarriers, including poor size homogeneity, inaccurate cargo loading, batch‐to‐batch variance, and unpredictable release kinetics.^10^, ^11^ In contrast, a mild emulsification method that uses a microfluidic device has been developed recently to produce emulsion droplets with an extremely narrow size distribution.^12^, ^13^ Combined with solidification methods, the droplets can provide excellent templates for the synthesis of advanced microcarriers with controlled size and structure and versatile compositions.^14^, ^15^

Generally, the microcarriers used as drug delivery systems are mainly designed based on passive mechanism, whereas intelligent microcarriers are rarely reached which can actively release encapsulated contents triggered by a noninvasive external stimulus.^16^, ^17^ Therefore, we designed a new type of near‐infrared (NIR) light‐responsive microcarriers for controllable drug delivery which was composed of graphene oxide (GO), poly (*N*‐isopropylacrylamide) (PNIPAM), and gelatin methacrylate (GelMA). Because of the low toxicity, abundant functional groups, and large specific surface area, GO has attracted extensive attentions in drug delivery field. In addition, as a kind of NIR‐absorbing nanomaterials, GO shows a high photothermal conversion efficiency in the NIR region.^18^, ^19^ PNIPAM is an outstanding thermoresponsive hydrogel, which undergoes a volume phase transition at the lower critical solution temperature (LCST) around 32 °C. At the temperature higher than LCST, the hydrophobicity of PNIPAM increases, resulting in volume shrinkage and water extrusion.^20^ GelMA has been widely used for various biomedical applications because of its inherent bioactivity and physicochemical tailorability.^21^ Though each of these three materials has been reported as drug delivery systems, the advantages of GO/PNIPAM/GelMA hybrid microcarriers with photothermal responsiveness for active drug release has not been explored.

**Figure 1:**
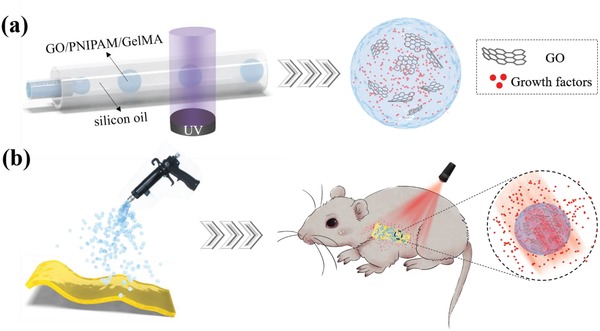
a) The schematic of the capillary microfluidic method for generating PDMs. b) The generated PDMs were sprayed onto a biological mesh and implanted into a rat for repairing tissue defects.

Here, we used microfluidic emulsion templates to generate photoresponsive delivery microcarriers (PDMs) by embedding GO into PNIPAM and GelMA hydrogels, as illustrated in **Figure** **1**. The GO could convert absorbed NIR light into thermal energy, increase the temperature of microcomposites, and trigger the shrinkage of PNIPAM chains. The addition of GelMA could not only improve the biocompatibility of this system, but also regulate the volume reduction of the microcarriers in response to NIR irradiations. It was demonstrated that the release kinetics of biomacromolecules from PDMs could be well‐controlled by turning the NIR light on and off. Also, in order to confirm the advantages of PDMs in future clinical application of tissue repair, we integrated the microcarriers with a biological mesh and applied the designed mesh into the regeneration process of a defected tissue. Because of the deep penetration of NIR light and active responsiveness of PDMs, we could regulate the release dosage of VEGFs in vivo by applying NIR irradiations in vitro. Therefore, the actives could be delivered at a demanded rate according to the practical conditions of defected tissues under minimal invasions. These features indicated that the proposed PDMs possessed of promising potentials in biomedical engineering.

## 2 Results and Discussion

**Figure 2:**
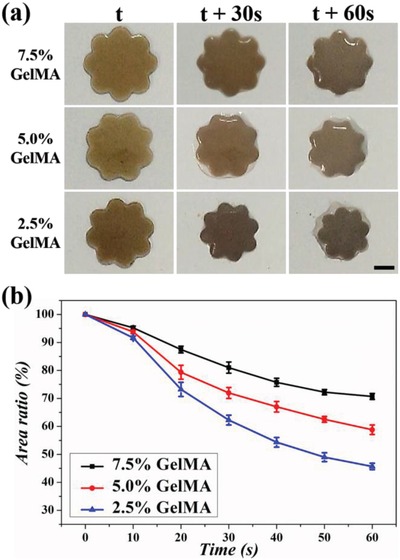
a) The thermoresponsive behaviors of the petal‐shaped hybrid hydrogels on heating stage when the temperature is set as 40 °C; the scale bar represents 300 µm. b) The area alterations of the hybrid hydrogels at different GelMA concentrations (*n* = 3 for each group); the error bar represents the standard deviation.

The stimuli‐responsive behaviors and cytocompatibility of GO/PNIPAM/GelMA hybrid hydrogels were first investigated and optimized. As a well‐known thermoresponsive polymer, PNIPAM becomes opaque and shrinks immediately at the same time when heated above the LCST. It returns to the initial state when the temperature is reduced below the transition point.^20^ In order to illustrate the thermoresponsive behaviors of the hybrid hydrogels, the petal‐shaped film was fabricated to observe the change of size more intuitively. As shown in **Figure** **2**a, when putting onto the heating stage (the temperature was set as 40 °C), the film shrank obviously, along with exclusion of water and deepening of color. The fast change in opacity and size indicated a well temperature responsiveness of the hybrid hydrogels, which is critical for intelligent drug delivery. It was also found that the concentration of GelMA could influence the temperature responsiveness of the hybrid hydrogels. As counted in Figure 2b, the shrinkage degree of the film decreased with the increasing concentration of the GelMA hydrogel. The reason for this phenomenon was that the hydrophilic intrinsic quality of GelMA would not be affected by temperature changes and thus it could restrain the hydrophobic alteration of the hybrid hydrogels when heated on the stage.

**Figure 3:**
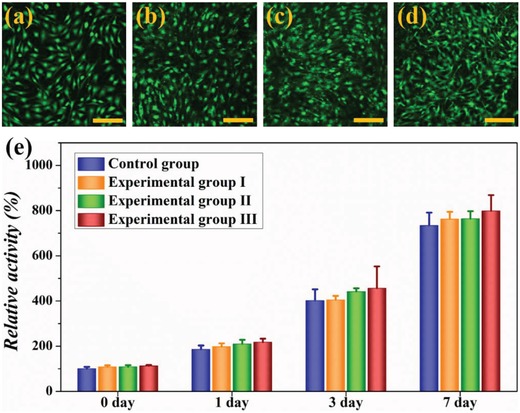
The fluorescent images of 3T3 cells cultured on a) blank wells, or hybrid hydrogels consisting of b) 2.5% GelMA, c) 5.0% GelMA, and d) 7.5% GelMA; the scale bar represents 50 µm. e) The CCK‐8 results of 3T3 cells in each group (*n* = 5); the error bar represents standard deviation.

The in vitro test results of cytocompatibility and safety are crucial for the next in vivo animal experiments.^22^ Here, the 3T3 cells were cultured with the hybrid hydrogels consisting of 2.5% (experimental group I), 5.0% (experimental group II), and 7.5% (experimental group III) GelMA, respectively, and the cells cultured in blank well were set as control group. As shown in **Figure** **3**a, the 3T3 cells already distributed on the surface of blank well homogenously after 24 h of culture. On the hybrid hydrogels, the cells appeared at a slightly higher confluency and distributed even in the depth of hydrogels (Figure 3b–d). The cells in all groups had a normal and healthy morphology. The results of CCK‐8 assay (Figure 3e) showed the consistent results with the cell fluorescent images. We could find that the cells in all experimental groups remained the same favorable metabolic activity as control group over the time course of this study. The excellent cytocompatibility of the hybrid hydrogels could attribute to the incorporation of GelMA which provided bioactive sequences (such as arginine‐glycine‐aspartic acid peptide) for cellular adhesion, proliferation, and differentiation.^23^ However, the cellular activity did not differ obviously when culturing with the hybrid hydrogels comprising different GelMA concentrations ranging from 2.5 to 7.5 wt%. Therefore, considering the cytocompatibility and thermoresponsive efficiency of the hybrid hydrogels, the concentration of GelMA was set as 2.5 wt% in the following experiments.

**Figure 4:**
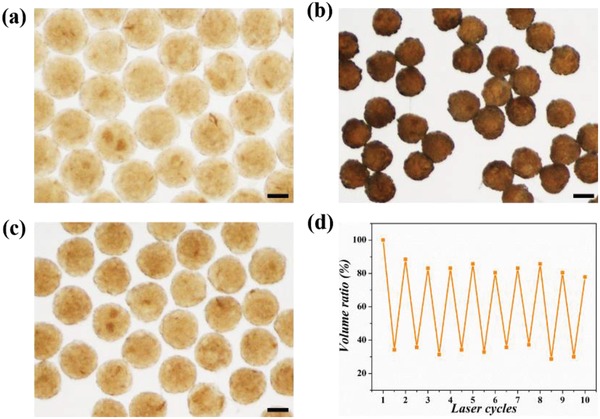
a) The photograph of PDMs under optical microscope. b) The shrunken PDMs under NIR irradiation. c) The PDMs return to their original swollen state when the NIR light is switched off. d) The volume variation of PDMs under ten cycles of NIR light on/off. The scale bar represents 100 µm.

After the above‐mentioned optimizing process, GO/PNIPAM/GelMA PDMs were prepared by photopolymerization of composite droplets generated using a simple capillary microfluidic device. First, the aqueous solution (0.2 wt% GO, 10 wt% PNIPAM, and 2.5 wt% GelMA) as the dispersed phase and the oily solution (silicon oil) as the continuous phase were respectively injected into the inlets of microfluidic device. As shown in Figure S1a in the Supporting Information, the co‐flow design of microfluidic device was optimized for generating monodisperse droplets by shearing the dispersed phase without addition of any surfactant. Then, after polymerizing the monomer in aqueous droplets in situ by ultraviolet (UV) exposure, the obtained microcarriers showed a narrow size distribution with a coefficient of variation (CV) below 5% and well‐defined sphericity (**Figure** **4**a; Figure S1b, Supporting Information). Previous studies pointed out that the poor monodispersity of the fabricated microcarriers from traditional techniques (CV > 10%) would cause their instability in physicochemical properties and limit their ability to control the drug‐release kinetics.^14^, ^24^ From the results of our experiment, it was demonstrated that microfluidics could generate microcarriers with high uniformity, which met the basic requirements for future biomedical applications. In addition, because of the excellent capacity of fluid manipulation in microfluidics, the diameters of microcarriers were precisely regulated by altering the flow rates of dispersed or continuous phases. As shown in Figure S1c,d in the Supporting Information, the diameters of generated microcarriers increased with the increasing inner flow rates, while they decreased when the outer flow rates increased.

Prior to developing the NIR‐responsive drug delivery system, it is vital to assess the volume‐shrinkage ability of the microcarriers under photothermal effect. For this purpose, we employed a focused NIR laser with a wavelength of 808 nm at a constant power of 1 W cm^−2^ on PDMs, and recorded the NIR‐responsive behavior by optical microscope. As shown in Figure 4b, the PDMs contracted obviously to ≈30% of their original volume. The reason of this phenomenon was that a nonradiative decay could excite GO to generate local heat under NIR treatment, and the PNIPAM hydrogel exhibited an immediate thermally induced phase transition.^25^, ^26^ When the NIR laser was switched off, the PDMs returned to their original swollen state (Figure 4c). In addition, the PDMs had good photothermal stability and repeatable responsiveness even after ten cycles of laser on/off (Figure 4d). The reversible volume‐shrinkage ability of the microcarriers could expel the absorbed water constantly, thus resulting in an active release of encapsulated drugs.

**Figure 5:**
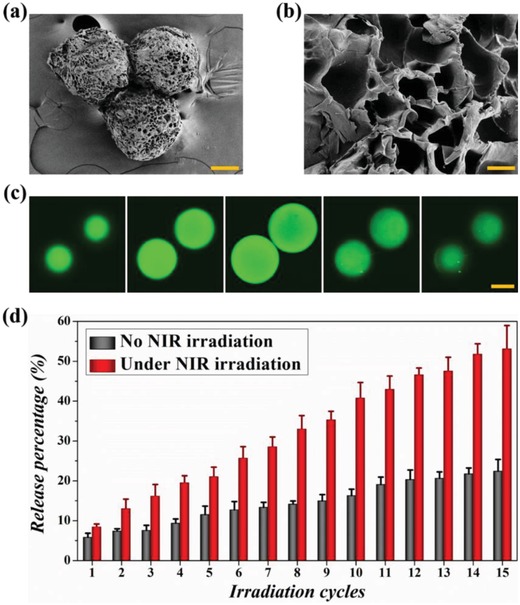
a) The whole view of PDMs under SEM; the scale bar represents 50 µm. b) The surface magnification view of PDMs under SEM; the scale bar represents 10 µm. c) The layer‐by‐layer photographs of PDMs obtained from CLSM; the scale bar represents 100 µm. d) The cumulative FITC‐BSA release from PDMs with or without repeated NIR irradiation (*n* = 3 for each group); the error bar represents standard deviation.

The scanning electron microscope (SEM) images of PDMs are shown in **Figure** **5**a,b, and we could find that numerous large pores spread all over the microcarriers. This porous structure provided sufficient spaces for PDMs to vigorously contract under NIR irradiation. As is well‐known, the drug distribution inside microcarriers significantly influence the release kinetics, and a uniform distribution can effectively avoid undesired burst release.^27^ To observe the protein molecules distribution in PDMs fabricated by microfluidics, fluorescein isothiocyanate‐bull serum albumin (FITC‐BSA) was loaded, and the layer‐by‐layer images of PDMs were obtained using a confocal laser scanning microscope (CLSM). As shown in Figure 5c, the microcarriers exhibited a uniform distribution of green fluorescence emitted from FITC‐BSA, which mainly contributed to the mild emulsification process afforded by microfluidics.

In order to evaluate the feasibility of using NIR light to trigger protein molecules releasing from PDMs, the FITC‐BSA cumulative release profiles of PDMs with or without repeated NIR irradiation (once every hour) were investigated. As shown in Figure 5d, the PDMs without NIR irradiation showed a low initial release of 5.8% in the first hour, followed by a sustained slow release over the next few hours, and the total release percentage at the 15th hour was 22.4%. In contrast, the PDMs with NIR irradiation exhibited an initial release of 8.5% in the first cycle, followed by an accelerated release over the next few cycles, and the total release percentage after 15 cycles reached at 53.1%. Therefore, in the absence of NIR light, the protein molecules could only release from PDMs at a very slow rate under diffusive force. However, when the NIR light was turned on, the photothermal effect of GO could heat the microcarriers and cause them to contract, resulting in the accelerated release of encapsulated actives. After the NIR light was switched off, the PDMs cooled down gradually and swelled to their initial state to prepare for the next triggered release.

Furthermore, to simulate the actual situation of drug administration in clinic, we divided PDMs into three experimental groups, receiving NIR irradiation once, twice, and thrice a day, respectively, and set PDMs not receiving NIR irradiation as control group. The cumulative release curves from all four groups are detailed in Figure S2 in the Supporting Information. It was obvious that as the frequency of NIR irradiation increased, the release rate of encapsulated actives accelerated accordingly. It took more than 11 days for control group to achieve a release percentage of 50%, while under NIR irradiation, the same release amount was achieved in less than 5 days. These results demonstrated that by applying the NIR light and regulating the irradiation frequency, the release rate of loaded actives from PDMs could be well‐tailored to achieve desired effect.

**Figure 6:**
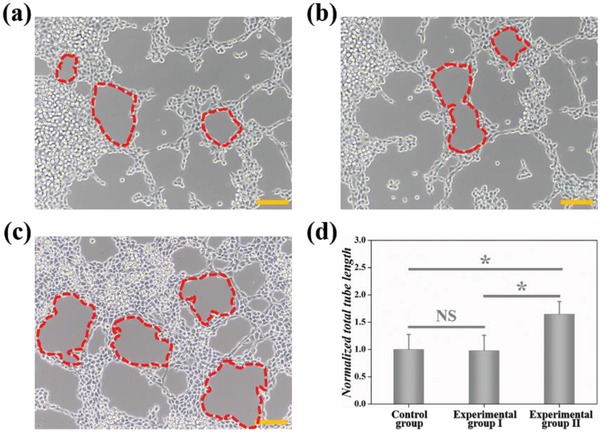
Optical microscopic image of HUVECs cultured in a) control group, b) experimental group I, and c) experimental group II. The red dotted area refers to typical tubular structures, and the scale bar represents 50 µm. d) Statistical analysis of total tube length in each group (*n* = 4); the error bar represents standard deviation. * *p* < 0.05, NS: not significant.

VEGFs, a kind of strong mitogens for ECs, can specially bind to their receptors on cell membrane, and promote the proliferation/migration of ECs to form blood vessels.^6^, ^7^ To investigate whether the biological activity of VEGFs was well‐preserved after being loaded into PDMs, we performed the tube formation analysis using human umbilical vein ECs (HUVECs), which was one of the most common methods to evaluate the angiogenic effects of actives. As shown in **Figure** **6**a,b, the HUVECs cultured in blank medium (control group) and medium containing unloaded PDMs (experimental group I) developed a small number of tubular structures. While the HUVECs cultured in medium containing VEGFs‐PDMs (experimental group II) developed a cloud of tubular structures (Figure 6c). From the statistical analysis in Figure 6d, we could see that the total tube length in experimental group II was much higher than that in control group and experimental group I (*p* < 0.05). These in vitro results indicated that the pro‐angiogenic of VEGFs was well‐preserved in PDMs. Under NIR irradiation, the microcarriers could expel VEGFs in surrounding medium to reach effective concentrations for inducing angiogenesis.

The repairing of tissue defects is a complex process that greatly depends on the extent and mechanism of injury. A key accompanying activity in the regeneration stage is the formation of new blood vessels which can provide adequate nutrients and oxygen for defected tissues.^4^, ^5^ The administration of GFs is a very common method to construct effective vascular networks. Previous studies have demonstrated that a controllable drug delivery system is necessary for realizing this strategy, since bolus injection of biomacromolecules generally lead to a poor outcome because of the high diffusibility and short half‐life time of GFs.^28^, ^29^ However, GFs are usually embedded into “unintelligent” drug‐carriers, and only released under passive diffusion or along with biomaterial degradation. This process is not well‐controlled and cannot regulate the release rate of GFs according to the individual's conditions. Therefore, an effective microcarrier for on‐demand administration of drugs should provide accumulation of scheduled drug amounts to desired body regions as a response to a noninvasive external stimulus.^30^ In the present study, we generated a novel kind of smart microcarriers, and the in vitro experiments demonstrated that the PDMs could respond to NIR irradiation, shrink rapidly, and release biomacromolecules accordingly.

**Figure 7:**
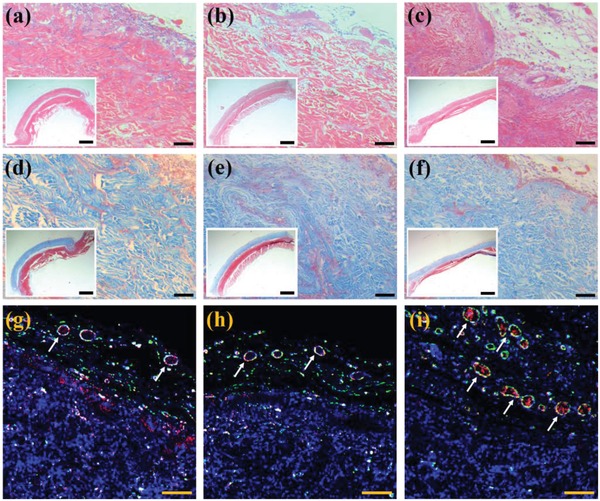
H&E stained images of samples repaired with a) PADM, b) PDMs‐PADM, and c) VEGFs‐PDMs‐PADM; the scale bar represents 100 µm. The lower inset shows the whole view of H&E stained sample; the scale bar represents 2 mm. Masson stained images of samples repaired with d) PADM, e) PDMs‐PADM, and f) VEGFs‐PDMs‐PADM; the scale bar represents 100 µm. The lower inset shows the whole view of Masson stained sample; the scale bar represents 2 mm. α‐SMA and CD31 double immunofluorescence stained images of samples repaired with g) PADM, h) PDMs‐PADM, and i) VEGFs‐PDMs‐PADM. The white arrowhead refers to mature vascular structures, and the scale bar represents 50 µm.

To assess the potential feasibility and regenerative capacity of PDMs, we established animal models of abdominal wall defects (AWDs) in rats randomized into 3 groups (Figure S3, Supporting Information). The rats received treatment with either porcine acellular dermal matrix (PADM), PADM containing unloaded PDMs (PDMs‐PADM), or PADM containing VEGFs‐PDMs (VEGFs‐PDMs‐PADM). After successful implantation, we first examined whether the external NIR irradiation could trigger photothermal effect of PDMs in vivo. By using an uncooled handheld infrared camera, the fluorescent images of rats' ventrolateral abdominal walls were recorded in Figure S4 in the Supporting Information. It was found that under NIR irradiation, the defected sites repaired with PDMs‐PADM and VEGFs‐PDMs‐PADM have a markedly elevated temperature than those repaired with PADM. The results suggested that the photothermal effect of PDMs could be reserved with negligible attenuation in the animal body, because of the strong capacity of NIR laser to penetrate the whole skin.^31^ All animals survived without signs of infection or rejection after implantation. At the fourth week, we opened the skin incisions and observed the repair of tissue defects in each group. From the photographs in Figure S5 in the Supporting Information, it was found that the boundaries of PADM and PDMs‐PADM remained apparent, indicating their poor ability to integrate with surrounding abdominal wall. In contrast, the VEGFs‐PDMs‐PADM was totally covered by a layer of granulation tissue and its shape became obscure. In addition, we could find some vascular structures on the surface of this scaffold. Hematoxylin and eosin (H&E) and Masson staining techniques were also performed on the samples taken from the operative sites to assess the repair procedure. As shown in H&E stained images (**Figure** **7**a–c), mild cell infiltrations were observed in each scaffold, particularly in the defected regions adjacent to the native tissues. The inflammation score in Figure S6 in the Supporting Information indicated that the incorporation of PDMs did not augment foreign body reactions (*p* > 0.05). New collagen fiber deposition surrounding each scaffold was visible by Masson staining (Figure 7d–f), and the collagen deposition amount was not significantly different between the three treatment groups (*p* > 0.05, Figure S7, Supporting Information).

α‐Smooth muscle actin (α‐SMA) and CD31 double immunofluorescence staining was performed to assess the functional impact of VEGFs released from PDMs on neovascularization. Representative images from each scaffold are presented in Figure 7g–i, the CD31 structures (red) were surrounded by α‐SMA positive cells (green), and the spatial relationship between CD31 and α‐SMA staining indicated mature vascular formation. We could find that the PADM repaired samples had less blood vessel formation, indicating that the biological mesh was not pro‐angiogenic in nature. Likewise, the PDMs‐PADM repaired samples also had minimal neovascularization. On the contrary, the VEGFs‐PDMs‐PADM repaired samples showed enhanced blood vessel formation when compared to the other two scaffolds. From the statistical analysis in Figure S8 in the Supporting Information, the vessel density in VEGFs‐PDMs‐PADM repaired samples was more than twice as that in PADM and PDMs‐PADM repaired samples (*p* < 0.05). The results demonstrated that VEGFs‐PDMs could maintain their biological activity in vivo, and promote neovascularization in the implanted sites. The VEGFs are primarily responsible for regulating angiogenesis in both embryonic and adult development, and have also been shown to stabilize new blood vessel growth during wound healing.^6^, ^7^ However, the application value of VEGFs in clinical settings is limited, and the short duration of action may be one of the primary causes. Our results showed that VEGFs‐PDMs could obviously induce blood vessel formation in the implanted sites. On one hand, the encapsulation of VEGFs into microcarriers protected the biomacromolecules from denaturation and proteolytic degradation. On the other hand, the PDMs could control the release kinetics of their cargos according to the NIR stimuli over a long time period, thus promoting angiogenesis continuously during the procedure of tissue regeneration.

## 3 Conclusion

In conclusion, a new type of intelligent microcarriers for active drug delivery was fabricated from composite droplets generated in a water‐in‐oil microfluidic system through a photopolymerization method. The PDMs exhibited a uniform spherical morphology, a porous structure, and their sizes could be well‐controlled by regulating the flow rates of dispersed and continuous phases. In vitro experiments demonstrated that the PDMs possessed of a steady and repeatable responsive ability to NIR light. These microcarriers shrunk immediately under irradiation, and could return to their original swollen state after leaving the stimulus. It was also demonstrated that the NIR irradiation could trigger the drug release from PDMs. As the frequency of NIR irradiation increased, the release rate was accelerated accordingly. Furthermore, the goal of active VEGFs delivery in vivo was successfully realized after implanting PDMs in a rat model of AWDs, because of the powerful penetrability of NIR laser to biotic tissues. The results from animal experiments showed that the NIR‐radiated VEGFs‐PDMs could promote the formation of new blood vessels and accelerate the repairing process of tissue defects. These characteristics of PDMs indicated their promising potentials in smart drug delivery and tissue regeneration.

## 4 Experimental Section

*The Thermoresponsive Behavior of Hybrid Hydrogels*: 10 wt% *N*‐isopropylacrylamide (NIPAM, Sigma‐Aldrich, USA), 0.34 wt% *N*, *N*′‐methylenebis(acrylamide) (BIS, Sigma‐Aldrich, USA), 2.5 wt% GelMA (Aladdin, China), 0.2 wt% GO (Nanjing XF NANO Materials Tech Co., China), and 1 vol% photoinitiator 2‐hydroxy‐2‐methylpropiophenone (HMPP, Sigma‐Aldrich, USA) were dispersed in water homogeneously and polymerized under ultraviolet light at a distance of 10 cm for about 20 s with a petal‐shaped mask to fabricate petal‐shaped films. For investigating the thermoresponsive behavior of the hybrid hydrogels, the concentration of NIPAM, BIS, and GO were kept constant, while the concentration of GelMA changed from 2.5% to 5.0% to 7.5%. After putting the different films onto a heating stage and keeping the temperature at 40 °C, the area alteration was recorded by a digital camera.

*In Vitro Cytocompatibility Experiment*: NIH 3T3 cells (obtained from the Cell Bank of Chinese Academy of Sciences, China) were cultured in completed medium that was composed of high sugar Dulbecco's modified Eagle's medium (DMEM, Invitrogen, China), 10 vol% fetal bovine serum (FBS), and 1 vol% penicillin‐streptomycin (Pen‐Strep). The hybrid hydrogels with 2.5% (experimental group I), 5.0% (experimental group II), and 7.5% (experimental group III) GelMA were fabricated, respectively, sterilized under UV irradiation overnight, cut into wafers with a diameter of 5 mm, and put into the bottom of a 24‐well plate. The 3T3 cells were then trypsinized and seeded into the 24‐well plate with a density of 2 × 10^5^ cells per well. After 24 h of culture, the cells adhered on the hydrogels were stained by adding 1.0 µL Calcein‐AM (Molecular Probes Co.) in the culture medium. After incubation for 1 h at 37 °C, the samples were washed with phosphate buffered saline (PBS) three times and observed under CLSM (FV10i, Olympus, Japan). The cellular activities were evaluated using a Cell Counting Kit‐8 (CCK‐8, Keygen Biotech Co., China) according to the manufacturer's protocol. Briefly, the samples were treated with CCK‐8 diluted (1:10) in the culture medium for 3 h in the incubator. The mixture was then transferred into a 96‐well plate, and the absorbance was measured using a microplate reader (Synergy HT, BioTek, USA) at 450 nm. The blank well without hydrogels was regarded as control group. The number of replicates was 5.

*Microfluidic Fabrication of PDMs*: Figure 1 shows the schematic process of PDMs generation by microfluidic co‐flow channels. The microfluidic device consisted of two (one inner and one outer) round and a square glass capillary, and they were co‐axially assembled on a glass slide. The inner and outer capillaries were identical, and their inner and outer diameters were 580 µm and 1 mm, respectively. The inner capillary was tapered by a micropipette puller (Sutter Instrument Co., Novato) and sanded to reach an orifice of 200 µm. All the junctions of microfluidic devices were sealed with needles and epoxy adhesive. The aqueous phase comprised 10 wt% NIPAM, 0.34 wt% BIS, 2.5 wt% GelMA, 0.2 wt% GO, and 1 vol% HMPP. The oil phase was silicon oil. The two phases were filled into separate syringes and injected into the corresponding inlets of microfluidic device through polyethylene tubes. The flow rates were controlled by a syringe pump (PHD 2000, Harvard Apparatus). Monodisperse aqueous droplets were formed under the shearing force of oil phase. The experimental setup was placed on the sample stage of an inverted optical microscope to monitor the droplet formation. The UV light was used to photopolymerize the droplets passing through the outer capillary. The fabricated microcarriers were collected into centrifugal tubes containing 1 mL deionized water and then centrifuged to remove the oil phase. The morphology characteristics of PDMs were explored by an optical microscope and SEM (Ultra Plus, Zeiss).

*The NIR‐Responsiveness of PDMs*: A focused NIR laser with a wavelength of 808 nm at a constant power of 1 W cm^−2^ was employed to irradiate PDMs for 1 min. Then, the NIR laser was switched off, and the PDMs were left to return to their original state. After 30 min, the PDMs were again exposed to NIR light. The above process was repeated for ten cycles, and the morphologic alteration of PDMs was recorded by an optical microscope. An AOS Imaging Studio V3.4.2 software was used to measure the diameters of the microcarriers before and after NIR irradiation.

*In Vitro Drug Release Experiment*: To investigate the encapsulation characteristic of biomacromolecules in PDMs, 1 mg mL^−1^ FITC‐BSA was mixed with the aqueous phase of microfluidic system to fabricate FITC‐BSA loaded PDMs (FITC‐BSA‐PDMs). The CLSM was used to observe the green fluorescence in FITC‐BSA‐PDMs layer‐by‐layer. To investigate whether NIR irradiation could trigger FITC‐BSA release from PDMs, FITC‐BSA‐PDMs were divided into two groups and put into centrifugal tubes containing 1 mL deionized water. The experimental group received NIR irradiation for 1 min once an hour, while the control group did not receive NIR irradiation. The hourly release amount of FITC‐BSA from each group was determined by detecting the OD value of supernatant at 493 nm using a microplate reader. To investigate the relationship between NIR irradiation frequency and FITC‐BSA release rate, FITC‐BSA‐PDMs were divided into three experimental groups, and received NIR irradiation once, twice, and thrice a day, respectively. In addition, FITC‐BSA‐PDMs not receiving NIR irradiation were set as control group. The daily release amount of FITC‐BSA from all four groups was determined using the same method. The number of replicates was 3.

*In Vitro Tube Formation Experiment*: HUVECs were purchased from the Cell Bank of Chinese Academy. M199 medium (Gibco, US) with 10% FBS and 1% Pen‐Strep was used to culture HUVECs. 100 µL of growth factor‐reduced Matrigel (BD Bioscience) was added into a 48‐well plate, followed by gel under 37 °C for 1 h. 3 × 10^4^ HUVECs were seeded in each well. Blank culture medium (control group), culture medium containing unloaded PDMs (experimental group I), and culture medium containing VEGFs‐PDMs (experimental group II) were used to treat the cells, respectively. For experimental groups I and II, the microcarriers were irradiated under NIR light for 1 min. An optical microscope was employed to observe the tube formation following 12 h incubation. The total tube length was quantified using the Image J software, and the reported values were normalized to the control group. The number of replicates was 4.

*In Vivo Repairing Study*: All animal tests were carried out according to the guidelines approved by the Animal Ethics Committee of Jinling Hospital affiliated to Medical School of Nanjing University. Male Sprague‐Dawley rats about 250 g were purchased from the Model Animals Research Center of Nanjing University and used as animal models. The bilateral partial AWDs were created as previously reported to evaluate the role of VEGFs‐PDMs in vessel formation and tissue regeneration.^3^ The defects in abdonimal walls were square with a side length of 15 mm. Eighteen rats were randomly divided into three groups: 1) Group A, in which bilateral defects were repaired with PADM versus PDMs‐PADM; 2) Group B, in which bilateral defects were repaired with PADM versus VEGFs‐PDMs‐PADM; 3) Group C, in which bilateral defects were repaired with PDMs‐PADM versus VEGFs‐PDMs‐PADM. The scaffolds were filled in the defected sites, and the four corners were sutured. The repaired defects were exposed to NIR irradiation for 2 min once a day from the second day after surgery. During NIR irradiation, the temperature of rats was recorded by the uncooled handheld infrared camera (FLIR Systems AB, Sweden). At the fourth week after surgery, the rats were killed and the entire defects with adjacent normal tissues were taken out for further analysis.

The samples were fixed in 10% formalin for 2 days, dehydrated with a graded series of ethanol, and embedded in paraffin. After that, the sections were stained with hematoxylin and eosin and Masson according to the previous procedure. Pictures of different magnifications were obtained from an optical microscope. For immunofluorescence staining, the sections were incubated with α‐SMA (1:200, Abcam) and CD31 (1:200, Abcam) primary antibodies at 4 °C overnight. Afterward, the sections were washed and incubated with Alexa Fluor 488‐conjugated and Alexa Fluor 555‐conjugated secondary antibodies (1:200, Abcam). Finally, the sections were rinsed and mounted with 4, 6‐diamidino‐2‐phenylindole (DAPI) mounting medium to label nuclei. The fluorescent images were examined under CLSM. To calculate the inflammation score, collagen deposition amount, and density of new blood vessels, the sections from each scaffold were evaluated by two pathologists blinded to the experimental groups.

*Statistical Analysis*: All statistical analyses were conducted using SPSS 20.0 software. The count data were presented as mean ± standard deviation. The comparisons between each group were performed using Student's *t*‐test. The difference was considered to be statistically significant if *p* < 0.05.

## Conflict of Interest

The authors declare no conflict of interest.

## Supporting information

Supplementary
