# Photoresponsive Delivery Microcarriers for Tissue Defects Repair

**DOI:** 10.1002/advs.202202182

**Published:** 2022-05-25

**Authors:** Xin Zhao, Yuxiao Liu, Changmin Shao, Min Nie, Qian Huang, Jieshou Li, Lingyun Sun, Yuanjin Zhao

*Adv. Sci*. **2019**, *6*, 1901280

DOI: 10.1002/advs.201901280

In the originally published article there is an error in Figure 6(b). The correct Figure 6 is reproduced below. This error does not affect the results or conclusions of this article. The authors apologize for any inconvenience this may have caused. This correction has been approved by all coauthors.

**Figure 6:**
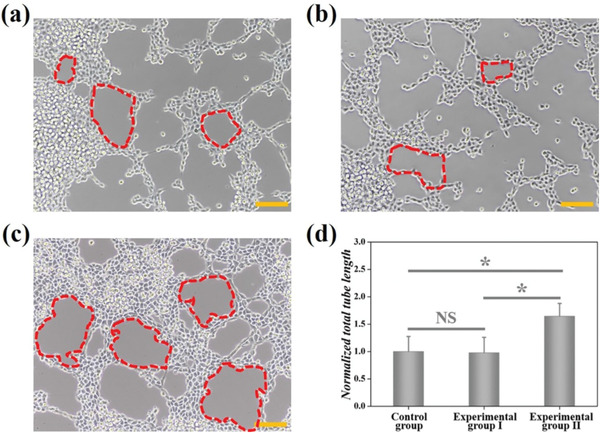
Optical microscopic image of HUVECs cultured in a) control group, b) experimental group I, and c) experimental group II. The red dotted area refers to typical tubular structures, and the scale bar represents 50 µm. d) Statistical analysis of total tube length in each group (n = 4); the error bar represents standard deviation. * p < 0.05, NS: not significant.

