# Supplementary material for: Photoresponsive Delivery Microcarriers for Tissue Defects Repair
Source: Adv Sci (Weinh). 2019 Aug 20;6(20):1901280. doi: 10.1002/advs.201901280 (PMC6794614; doi:10.1002/advs.201901280)
Supplement: Supplementary file 1 — Supplementary [file ADVS-6-1901280-s001.pdf]

Copyright WILEY-VCH Verlag GmbH & Co. KGaA, 69469 Weinheim, Germany, 2019.

Supporting Information

for *Adv. Sci.,* DOI: 10.1002/advs.201901280

Photoresponsive Delivery Microcarriers for Tissue Defects Repair

Xin Zhao, Yuxiao Liu, Changmin Shao, Min Nie, Qian Huang, Jieshou Li, Lingyun Sun,* and Yuanjin Zhao**

Copyright WILEY-VCH Verlag GmbH & Co. KGaA, 69469 Weinheim, Germany, 2019.

Supporting Information

Photo-responsive delivery microcarriers for tissue defects repair

Xin Zhao, Yuxiao Liu, Changmin Shao, Min Nie, Qian Huang, Jieshou Li, Lingyun Sun* and Yuanjin Zhao**

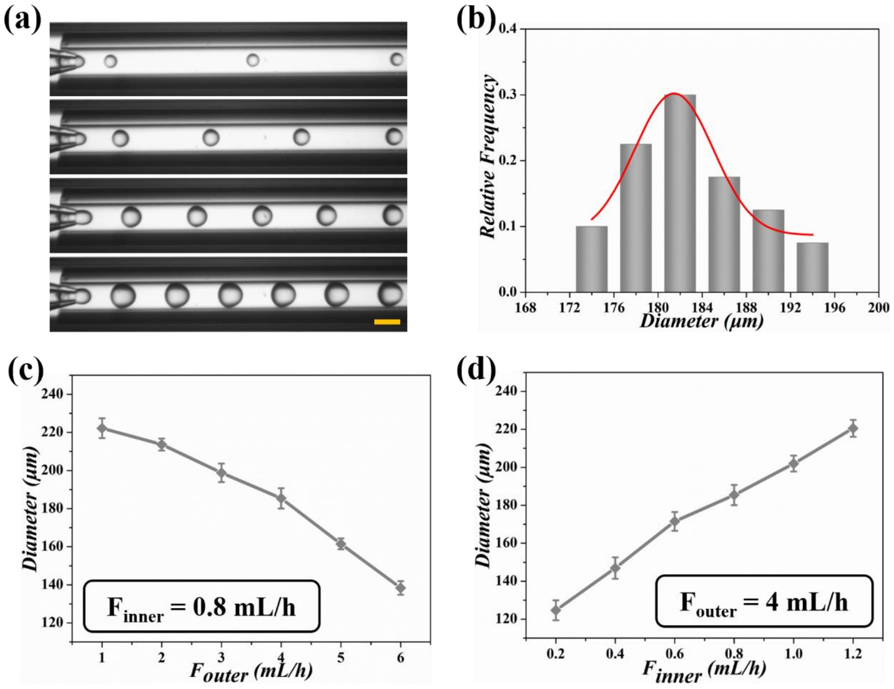

Figure S1. (a) Real-time generation image of GO/PNIPAM/GelMA droplets in the microfluidic device with different flow rates of inner and outer phases, the scale bar represents 600 μm. (b) The diameter distribution of generated PDMs (n = 40). (c) The relationship between the diameters of PDMs and the outer flow rates (n = 12 at each outer flow rate). (d) The relationship between the diameters of PDMs and the inner flow rates (n = 12 at each inner flow rate). The error bar represents standard deviation.

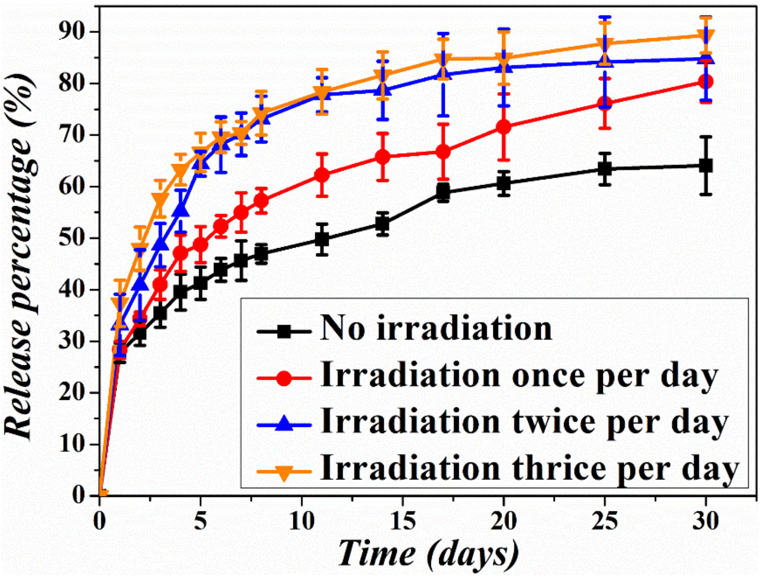

Figure S2. In vitro accumulative FITC-BSA release from PDMs under different frequency of NIR irradiation (n = 3 for each group), the error bar represents standard deviation.

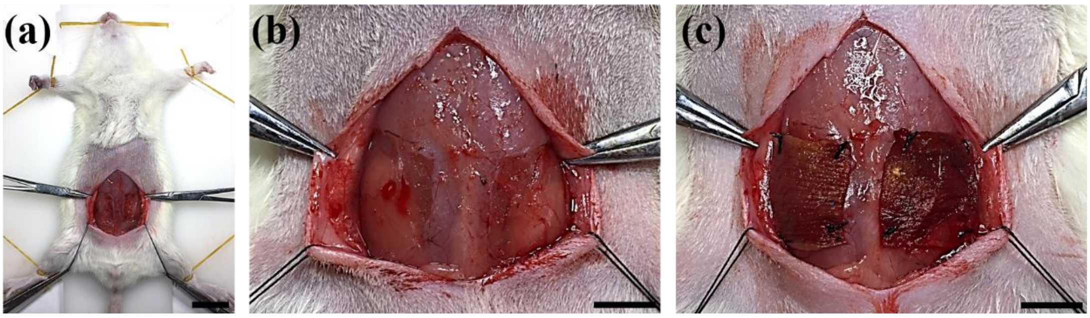

Figure S3. (a) The establishment of a rat model of bilateral ventrolateral partial thickness AWDs, the scale bar represents 3 cm. (b) The magnified photograph of operative region, the scale bar represents 1 cm. (c) Different scaffolds are fixed to the defect sites, the scale bar represents 1 cm.

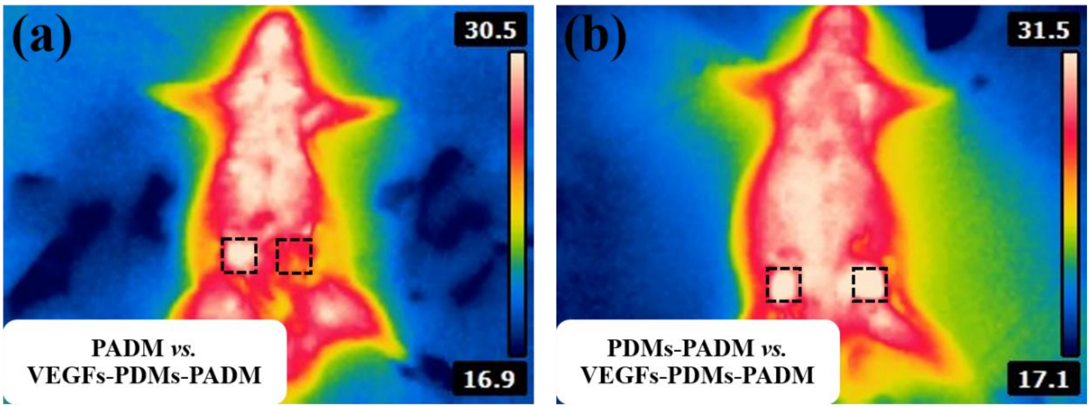

Figure S4. Fluorescent images of rats' ventrolateral abdominal walls under NIR irradiation.

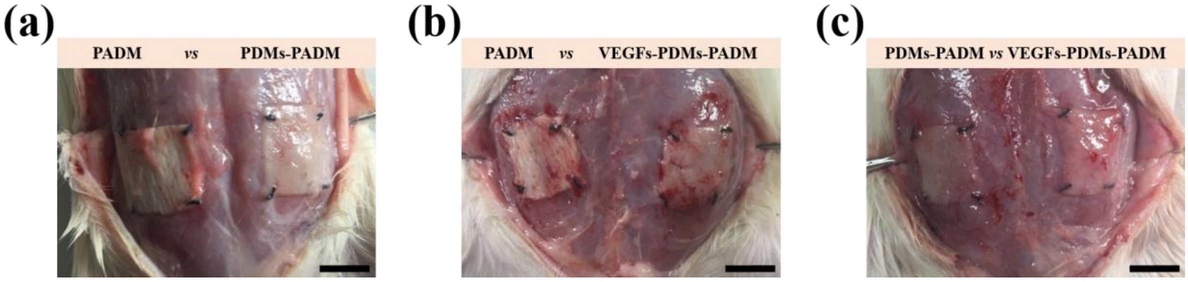

Figure S5. Gross observations of the AWDs repaired with different scaffolds at four weeks after implantation, the scale bar represents 1 cm.

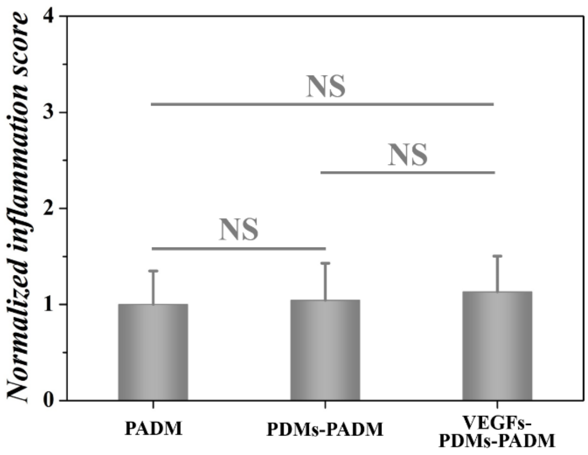

Figure S6. Statistical analysis of the inflammation score in each scaffold (n = 12). NS not significant.

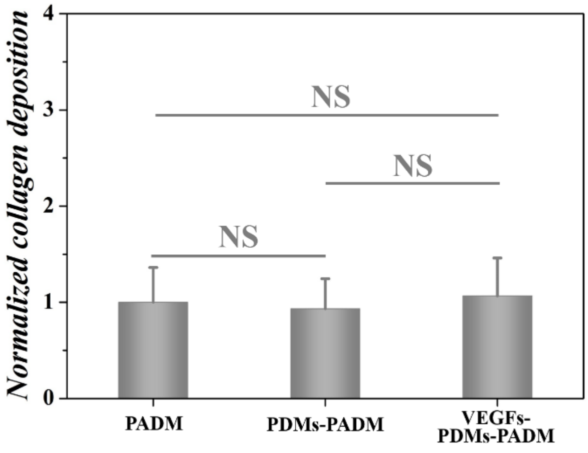

Figure S7. Statistical analysis of the collagen deposition in each scaffold (n = 12). NS not significant.

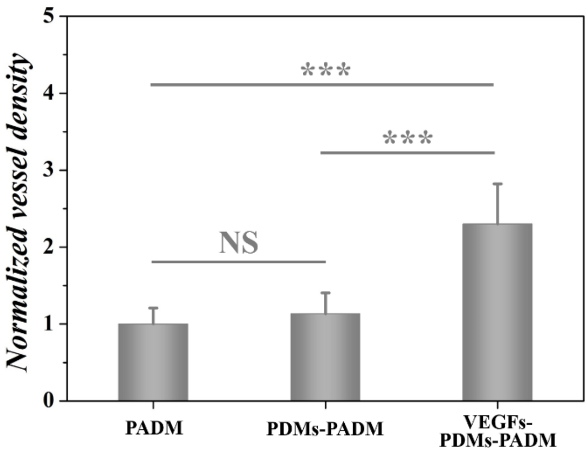

Figure S8. Statistical analysis of the vessel density in each scaffold (n = 12). *** $p < 0.001$, NS not significant.
